# Publisher’s Note: We Changed Page Numbers to Article Numbers for Articles Published in *Pediatric Reports* Volume 1–Volume 12, Issue 2

**DOI:** 10.3390/pediatric14010014

**Published:** 2022-02-23

**Authors:** Pediatric Reports Editorial Office

**Affiliations:** MDPI AG, St. Alban-Anlage 66, 4052 Basel, Switzerland; pediatrrep@mdpi.com

*Pediatric Reports* [1] was published by PAGEPress from Volume 1 (2009) to Volume 12, Issue 2 (2020). From Volume 12, Issue 3 (2020), *Pediatric Reports* is published by MDPI.

Previous articles in Volume 1–Volume 12, Issue 2, published by PAGEPress in Open Access under a CC-BY (or CC-BY-NC-ND) licence, are now hosted by MDPI on mdpi.com as a courtesy and upon agreement with PAGEPress.

To standardize the metadata format of all previous articles, MDPI republished 245 articles of Volume 1–Volume 12, Issue 2 with article numbers replacing page numbers (Table 1). MDPI also corrected Issue Number errors that occurred during importing of batch data for 24 articles in Volumes 1–5 and 7 (Table 2).

## Figures and Tables

**Table 1:** MDPI changed page numbers to articles numbers for 275 articles in Volume 1–Volume 12, Issue 2.

DOI	Previous Page Number	Current Article Number
10.4081/pr.2009.e1	1–9	e1
10.4081/pr.2009.e2	10–12	e2
10.4081/pr.2009.e3	13–15	e3
10.4081/pr.2009.e4	16–18	e4
10.4081/pr.2009.e6	20–22	e6
10.4081/pr.2009.e7	23–26	e7
10.4081/pr.2009.e8	27–29	e8
10.4081/pr.2009.e9	30–31	e9
10.4081/pr.2010.e1	1–8	e1
10.4081/pr.2010.e2	9–10	e2
10.4081/pr.2010.e3	11–14	e3
10.4081/pr.2010.e4	15–17	e4
10.4081/pr.2010.e5	18–19	e5
10.4081/pr.2010.e6	20–21	e6
10.4081/pr.2010.e7	22–25	e7
10.4081/pr.2010.e8	26–28	e8
10.4081/pr.2010.e9	29–31	e9
10.4081/pr.2010.e10	32–35	e10
10.4081/pr.2010.e11	36–37	e11
10.4081/pr.2010.e12	38–41	e12
10.4081/pr.2010.e13	42–43	e13
10.4081/pr.2010.e14	44–47	e14
10.4081/pr.2010.e15	48–50	e15
10.4081/pr.2010.e16	51–54	e16
10.4081/pr.2010.e17	55–57	e17
10.4081/pr.2010.e18	58–61	e18
10.4081/pr.2010.e19	62–66	e19
10.4081/pr.2010.e20	67–68	e20
10.4081/pr.2011.e1	1–5	e1
10.4081/pr.2011.e2	6–8	e2
10.4081/pr.2011.e3	9–10	e3
10.4081/pr.2011.e4	11–12	e4
10.4081/pr.2011.e5	13–16	e5
10.4081/pr.2011.e6	17–20	e6
10.4081/pr.2011.e7	21–24	e7
10.4081/pr.2011.e8	25–26	e8
10.4081/pr.2011.e9	27–30	e9
10.4081/pr.2011.e10	31–36	e10
10.4081/pr.2011.e11	37–38	e11
10.4081/pr.2011.e12	39–41	e12
10.4081/pr.2011.e13	42–44	e13
10.4081/pr.2011.e14	45–48	e14
10.4081/pr.2011.e15	49–50	e15
10.4081/pr.2011.e16	51–64	e16
10.4081/pr.2011.e17	65–78	e17
10.4081/pr.2011.e18	79–80	e18
10.4081/pr.2011.e19	81–83	e19
10.4081/pr.2011.e20	84–86	e20
10.4081/pr.2011.e21	87–90	e21
10.4081/pr.2011.e22	91–92	e22
10.4081/pr.2011.e23	93–96	e23
10.4081/pr.2011.e24	97–99	e24
10.4081/pr.2011.e26	103–107	e26
10.4081/pr.2011.e27	108–110	e27
10.4081/pr.2011.e28	111–113	e28
10.4081/pr.2011.e29	114–118	e29
10.4081/pr.2011.e30	119–121	e30
10.4081/pr.2011.e31	122–124	e31
10.4081/pr.2011.e32	125–126	e32
10.4081/pr.2011.e33	127–128	e33
10.4081/pr.2011.e34	129–132	e34
10.4081/pr.2011.s2.e1	1–2	e1
10.4081/pr.2011.s2.e2	3	e2
10.4081/pr.2011.s2.e3	4	e3
10.4081/pr.2011.s2.e4	5–7	e4
10.4081/pr.2011.s2.e5	8–10	e5
10.4081/pr.2011.s2.e6	11–13	e6
10.4081/pr.2011.s2.e7	14–17	e7
10.4081/pr.2011.s2.e8	18–20	e8
10.4081/pr.2011.s2.e9	21–24	e9
10.4081/pr.2011.s2.e10	25–27	e10
10.4081/pr.2011.s2.e11	28–31	e11
10.4081/pr.2011.s2.e12	32–33	e12
10.4081/pr.2011.s2.e13	34–37	e13
10.4081/pr.2011.s2.e14	38–42	e14
10.4081/pr.2011.s2.e15	43–47	e15
10.4081/pr.2012.e1	1–4	e1
10.4081/pr.2012.e2	5–9	e2
10.4081/pr.2012.e3	10–11	e3
10.4081/pr.2012.e4	12–13	e4
10.4081/pr.2012.e5	14–16	e5
10.4081/pr.2012.e6	17–20	e6
10.4081/pr.2012.e7	21–24	e7
10.4081/pr.2012.e8	25–29	e8
10.4081/pr.2012.e9	30–33	e9
10.4081/pr.2012.e10	34–37	e10
10.4081/pr.2012.e11	38–43	e11
10.4081/pr.2012.e12	44–47	e12
10.4081/pr.2012.e13	48–51	e13
10.4081/pr.2012.e14	52–53	e14
10.4081/pr.2012.e15	54–56	e15
10.4081/pr.2012.e16	57–63	e16
10.4081/pr.2012.e17	64–67	e17
10.4081/pr.2012.e18	68–69	e18
10.4081/pr.2012.e19	70–77	e19
10.4081/pr.2012.e20	78–81	e20
10.4081/pr.2012.e21	82–84	e21
10.4081/pr.2012.e22	85	e22
10.4081/pr.2012.e23	86–87	e23
10.4081/pr.2012.e24	88–90	e24
10.4081/pr.2012.e25	91–93	e25
10.4081/pr.2012.e26	94–96	e26
10.4081/pr.2012.e27	97	e27
10.4081/pr.2012.e28	98–100	e28
10.4081/pr.2012.e29	101–104	e29
10.4081/pr.2012.e30	105–107	e30
10.4081/pr.2012.e31	108–111	e31
10.4081/pr.2012.e32	112–114	e32
10.4081/pr.2012.e33	115–116	e33
10.4081/pr.2012.e34	117–118	e34
10.4081/pr.2012.e35	119–123	e35
10.4081/pr.2012.e36	124–126	e36
10.4081/pr.2012.e37	127–129	e37
10.4081/pr.2013.e1	1–7	e1
10.4081/pr.2013.e2	8–12	e2
10.4081/pr.2013.e3	13–16	e3
10.4081/pr.2013.e4	17–19	e4
10.4081/pr.2013.e5	20–23	e5
10.4081/pr.2013.e6	24–27	e6
10.4081/pr.2013.e7	28–30	e7
10.4081/pr.2013.e8	31–34	e8
10.4081/pr.2013.e9	35–37	e9
10.4081/pr.2013.e10	38–42	e10
10.4081/pr.2013.e11	43–47	e11
10.4081/pr.2013.e12	48–49	e12
10.4081/pr.2013.e13	51–52	e13
10.4081/pr.2013.e14	53–57	e14
10.4081/pr.2013.e15	58–63	e15
10.4081/pr.2013.e16	64–72	e16
10.4081/pr.2013.e17	73–75	e17
10.4081/pr.2013.e18	76–80	e18
10.4081/pr.2013.e19	81–85	e19
10.4081/pr.2014.5090	26–28	5090
10.4081/pr.2014.5112	23–25	5112
10.4081/pr.2014.5118	1–7	5118
10.4081/pr.2014.5126	8–11	5126
10.4081/pr.2014.5160	31–32	5160
10.4081/pr.2014.5186	19–22	5186
10.4081/pr.2014.5194	12–15	5194
10.4081/pr.2014.5311	16–18	5311
10.4081/pr.2014.5332	29–30	5332
10.4081/pr.2014.5368	33–36	5368
10.4081/pr.2014.5447	37–39	5447
10.4081/pr.2014.5491	49–50	5491
10.4081/pr.2014.5532	40–43	5532
10.4081/pr.2014.5534	44–48	5534
10.4081/pr.2014.5596	51–52	5596
10.4081/pr.2014.5619	53–55	5619
10.4081/pr.2014.5660	56–60	5660
10.4081/pr.2015.5578	16–18	5578
10.4081/pr.2015.5615	7–12	5615
10.4081/pr.2015.5659	1–6	5659
10.4081/pr.2015.5682	13–15	5682
10.4081/pr.2015.5703	22–27	5703
10.4081/pr.2015.5760	19–21	5760
10.4081/pr.2015.5795	31–34	5795
10.4081/pr.2015.5817	74–75	5817
10.4081/pr.2015.5858	45–47	5858
10.4081/pr.2015.5859	28–30	5859
10.4081/pr.2015.5872	38–44	5872
10.4081/pr.2015.5873	51–53	5873
10.4081/pr.2015.5914	54–55	5914
10.4081/pr.2015.5928	62–64	5928
10.4081/pr.2015.5936	48–50	5936
10.4081/pr.2015.5938	56–58	5938
10.4081/pr.2015.5951	76–78	5951
10.4081/pr.2015.5955	35–37	5955
10.4081/pr.2015.5999	59–61	5999
10.4081/pr.2015.6056	65–70	6056
10.4081/pr.2015.6228	72–73	6228
10.4081/pr.2015.6338	79–80	6338
10.4081/pr.2016.6112	15–20	6112
10.4081/pr.2016.6159	1–3	6159
10.4081/pr.2016.6188	12–14	6188
10.4081/pr.2016.6370	21–26	6370
10.4081/pr.2016.6395	4–5	6395
10.4081/pr.2016.6456	9–11	6456
10.4081/pr.2016.6487	42–45	6487
10.4081/pr.2016.6488	30–33	6488
10.4081/pr.2016.6516	34–38	6516
10.4081/pr.2016.6517	39–41	6517
10.4081/pr.2016.6555	46–47	6555
10.4081/pr.2016.6576	68–70	6576
10.4081/pr.2016.6596	27–29	6596
10.4081/pr.2016.6613	74–76	6613
10.4081/pr.2016.6615	50–52	6615
10.4081/pr.2016.6626	48–49	6626
10.4081/pr.2016.6632	53–58	6632
10.4081/pr.2016.6643	61–62	6643
10.4081/pr.2016.6685	63–67	6685
10.4081/pr.2016.6885	71–73	6885
10.4081/pr.2017.6810	14–15	6810
10.4081/pr.2017.6946	1–3	6946
10.4081/pr.2017.6962	10–13	6962
10.4081/pr.2017.6973	7–9	6973
10.4081/pr.2017.6984	4–6	6984
10.4081/pr.2017.7045	33–35	7045
10.4081/pr.2017.7084	17–20	7084
10.4081/pr.2017.7085	50–52	7085
10.4081/pr.2017.7163	29–32	7163
10.4081/pr.2017.7211	26–28	7211
10.4081/pr.2017.7214	21–25	7214
10.4081/pr.2017.7227	70–77	7227
10.4081/pr.2017.7240	64–69	7240
10.4081/pr.2017.7266	36–39	7266
10.4081/pr.2017.7270	53–54	7270
10.4081/pr.2017.7284	42–46	7284
10.4081/pr.2017.7294	58–63	7294
10.4081/pr.2017.7300	40–41	7300
10.4081/pr.2017.7301	47–49	7301
10.4081/pr.2017.7305	55–57	7305
10.4081/pr.2017.7341	78–80	7341
10.4081/pr.2017.7489	84–87	7489
10.4081/pr.2018.7232	17–23	7232
10.4081/pr.2018.7339	1–7	7339
10.4081/pr.2018.7424	11–13	7424
10.4081/pr.2018.7500	29–32	7500
10.4081/pr.2018.7514	14–16	7514
10.4081/pr.2018.7604	24–25	7604
10.4081/pr.2018.7636	46–48	7636
10.4081/pr.2018.7648	33–36	7648
10.4081/pr.2018.7689	37–38	7689
10.4081/pr.2018.7705	28–42	7705
10.4081/pr.2018.7752	43–45	7752
10.4081/pr.2019.7386	6–10	7386
10.4081/pr.2019.7839	1–5	7839
10.4081/pr.2019.7848	31–34	7848
10.4081/pr.2019.7951	21–24	7951
10.4081/pr.2019.7953	13–14	7953
10.4081/pr.2019.7954	15–20	7954
10.4081/pr.2019.7973	11–12	7973
10.4081/pr.2019.7993	35–37	7993
10.4081/pr.2019.7997	25–30	7997
10.4081/pr.2019.8029	64–71	8029
10.4081/pr.2019.8161	41–43	8161
10.4081/pr.2019.8165	44–48	8165
10.4081/pr.2019.8166	56–58	8166
10.4081/pr.2019.8184	38–40	8184
10.4081/pr.2019.8190	49–52	8190
10.4081/pr.2019.8206	53–55	8206
10.4081/pr.2019.8224	78–80	8224
10.4081/pr.2019.8248	75–77	8248
10.4081/pr.2019.8250	72–74	8250
10.4081/pr.2019.8251	59–63	8251
10.4081/pr.2020.7405	3–6	7405
10.4081/pr.2020.7998	7–13	7998
10.4081/pr.2020.8231	20–21	8231
10.4081/pr.2020.8254	14–19	8254
10.4081/pr.2020.8352	35–38	8352
10.4081/pr.2020.8382	29–33	8382
10.4081/pr.2020.8430	42–45	8430
10.4081/pr.2020.8476	26–28	8476
10.4081/pr.2020.8483	39–41	8483
10.4081/pr.2020.8486	22–25	8486
10.4081/pr.2020.8495	1–2	8495
10.4081/pr.2020.8505	50–55	8505
10.4081/pr.2020.8535	56–60	8535
10.4081/pr.2020.8595	61–63	8595
10.4081/pr.2020.8690	1–3	8690
10.4081/pr.2020.8691	4–7	8691
10.4081/pr.2020.8692	8–10	8692
10.4081/pr.2020.8693	11–14	8693
10.4081/pr.2020.8694	15–17	8694
10.4081/pr.2020.8695	18–22	8695
10.4081/pr.2020.8696	23–26	8696
10.4081/pr.2020.8697	27–29	8697
10.4081/pr.2020.8698	30–33	8698
10.4081/pr.2020.8699	34–38	8699
10.4081/pr.2020.8700	39–43	8700
10.4081/pr.2020.8701	44–46	8701
10.4081/pr.2020.8702	47–51	8702
10.4081/pr.2020.8703	52–56	8703
10.4081/pr.2020.8758	1	8758
10.4081/pr.2020.8823	64–66	8823

**Table 2:** MDPI corrected Issue numbers for 24 articles in Volumes 1–5 and 7.

DOI	Previous Issue Number	Current Issue Number
10.4081/3929	13	s3
10.4081/4585	12	s2
10.4081/pr.2009.s1	11	s1
10.4081/pr.2010.s1	11	s1
10.4081/pr.2011.s1	11	s1
10.4081/pr.2011.s2.e1	12	s2
10.4081/pr.2011.s2.e2	12	s2
10.4081/pr.2011.s2.e3	12	s2
10.4081/pr.2011.s2.e4	12	s2
10.4081/pr.2011.s2.e5	12	s2
10.4081/pr.2011.s2.e6	12	s2
10.4081/pr.2011.s2.e7	12	s2
10.4081/pr.2011.s2.e8	12	s2
10.4081/pr.2011.s2.e9	12	s2
10.4081/pr.2011.s2.e10	12	s2
10.4081/pr.2011.s2.e11	12	s2
10.4081/pr.2011.s2.e12	12	s2
10.4081/pr.2011.s2.e13	12	s2
10.4081/pr.2011.s2.e14	12	s2
10.4081/pr.2011.s2.e15	12	s2
10.4081/2012.s1.abs	11	s1
10.4081/2012.s1.rel	11	s2
10.4081/2013.s1.abs	11	s1
10.4081/2013.s1.rel	11	s1
10.4081/pr.2015.6202	11	s1

## References

[1] Pediatric Reports Homepage. https://www.mdpi.com/journal/pediatrrep.

